# Women’s empowerment, household dietary diversity, and child anthropometry among vulnerable populations in Odisha, India

**DOI:** 10.1371/journal.pone.0305204

**Published:** 2024-08-06

**Authors:** Sylvester Ogutu, Jonathan Mockshell, James Garrett, Thea Ritter, Ricardo Labarta, Diego Alvarez, Swamikannu Nedumaran, Carolina Gonzalez, Elisabetta Gotor

**Affiliations:** 1 International Center for Tropical Agriculture (CIAT), Kampala, Uganda; 2 International Center for Tropical Agriculture (CIAT), Cali, Colombia; 3 Bioversity International, Rome, Italy; 4 Previously with the International Center for Tropical Agriculture (CIAT), Cali, Colombia; 5 The International Crops Research Institute for the Semi-Arid Tropics (ICRISAT), Hyderabad, India; University of Gour Banga, INDIA

## Abstract

Women’s empowerment has been promoted by researchers and development practitioners as one of the most promising strategies to address widespread hunger and malnutrition. However, the relationship between women’s empowerment and dietary diversity and child nutrition has rarely been studied among vulnerable populations or individuals at greater risk of poor physical and social health status. Moreover, the effects of different domains of women’s empowerment on nutritional outcomes, including dietary diversity and child anthropometry, have rarely been examined, especially with panel data. Using two rounds of panel data from 1900 households and fixed effects regression models, we analyze the effect of women’s empowerment on household dietary diversity score (HDDS) and child anthropometry among the particularly vulnerable tribal groups in Odisha, India. We also estimate the effects of various decision-making domains of women’s empowerment on HDDS and child anthropometry to understand which empowerment domains matter for nutrition. Results show that women’s empowerment is positively associated with HDDS (coef. 0.41 food groups; *p* < 0.1) and reduces the prevalence of underweight (coef. 39%; *p* < 0.05) and wasting (coef. 56%; *p <* 0.1) in children but has no effect on the prevalence of child stunting. Women’s empowerment in agricultural input use; output sales; income; food purchases; and credit, group membership, and employment contribute to improved dietary diversity and child nutrition. We conclude that women’s empowerment contributes to improved dietary diversity and child nutrition and is a promising strategy to improve farm household diets and child nutrition among vulnerable populations. Strengthening women’s empowerment through the promotion of women’s access to land and other agricultural inputs, market participation, access to information, capital, and credit is important.

## Introduction

Hunger and malnutrition are widespread across the globe. Recent statistics show that at least 720 million people are undernourished and about 2 billion people are micronutrient deficient [1]. Hunger and malnutrition contribute to increased cases of nutrition-related diseases, impaired growth and reduced cognitive ability in children, and deaths [2, 3]. The majority of people affected by hunger and malnutrition are smallholder farmers who mainly depend on agriculture for their livelihood [1, 4]. Women’s empowerment–equipping women with “the ability to make strategic life choices and access to resources, especially in settings where this ability has been denied” [5]–is a strategy that has been promoted by researchers and development practitioners to address hunger and malnutrition [6, 7].

Women’s empowerment is important for improved nutrition in smallholder farm households partly because women play a significant role in agricultural productivity and food and nutrition security. For instance, women account for at least 40% of agricultural labor in developing countries [8]. Yet, women are still disadvantaged compared to men in terms of asset ownership, agricultural input use, and other land and non-land resources that can improve agricultural productivity and food security [9–11]. Moreover, women are usually the primary caregivers. Therefore, their empowerment can affect the choice of food and investments in child nutrition, health, and education [12]. Lastly, resources are not often pooled and reallocated equally among household members. Instead, allocation typically depends on each individual’s bargaining power [13]. Thus, increased empowerment of women can improve their access to and control over resources in the household, and contribute to increased agricultural productivity and improved nutritional outcomes [8, 10, 14].

Women’s empowerment is conceptualized to improve nutritional outcomes of rural farm households through several avenues or pathways. The avenues include: (a) the effect of women’s participation in agriculture on their own health and nutritional status; (b) the effect of women’s participation in agriculture on their time allocation; and (c) improvement of women’s social status and through access to and control over resources [6]. However, the evidence supporting the strength of these mechanisms or pathways–especially in agriculture and nutrition-related programs–remains scarce [15, 16].

Previous studies have shown that women’s empowerment is positively associated with improved maternal and child nutrition [8, 12, 17–21], larger household budgetary allocations to food [22], and improved child nutrition and education [23]. However, the literature on the effects of women’s empowerment has several gaps. First, the relationship between women’s empowerment and household dietary diversity and child nutrition has rarely been studied among vulnerable populations or individuals at greater risk of poor physical and social health status [24]. Yet, it is important to study vulnerable populations as they often experience severe malnutrition problems when faced with shocks, such as droughts, sudden increase in agricultural input or food prices and loss of income earners [25]. Women and children have generally been considered vulnerable and have been studied, but studies focusing on populations with disparities in physical, economic, and social status when compared with the dominant populations are rare. Second, the effects of different domains of women’s empowerment on nutritional outcomes, including dietary diversity and child anthropometry, have rarely been examined, especially with panel data [24, 26]. To the best of our knowledge, only [14, 16, 24] have examined the effects of various domains of women’s empowerment on different nutritional outcomes. However, these studies rely on cross-sectional data which are limited for drawing robust causal inference. Previous studies mostly provide associational evidence as they used cross-sectional data which are limited for drawing robust causal inference [27].

We address these research gaps and provide several contributions to the literature. First, we evaluate the effects of women’s empowerment on HDDS, value of home-produced and consumed food per adult equivalent, and child anthropometric indicators. Second, we conduct this study in India among the Odisha particularly vulnerable tribal groups (details further below), adding to the evidence of the impact of agricultural interventions among vulnerable population groups. Third, besides analyzing the effects of an aggregated women’s empowerment index, we estimate the effects of the various decision-making domains (agricultural input use (production): sales, income, food purchases, and other (employment, access to credit, and group membership)) of the women’s empowerment index on the selected nutritional outcomes to understand which empowerment domains matter for nutrition. Lastly, as most previous studies rely on cross-sectional data and mainly provide associational evidence, we use panel data from two survey rounds and fixed effects regression models to rigorously analyze the effect of women’s empowerment.

The next section describes the study context. The materials and methods section describes the study area and sampling design; measurement of indicators of dietary diversity, child nutrition, and women’s empowerment; data analysis approach and ethical considerations. The results and discussion section presents the results of the analysis of the effects of women’s empowerment on nutritional outcomes. The last section summarizes the key findings and concludes.

## Study context

In spite of the considerable income growth, India continues to be the largest contributor to global prevalence of hunger and malnutrition. India was found to have serious levels of hunger and was ranked 101 out of 116 countries in the most recent Global Hunger Index, which is computed based on four component indicators, namely undernourishment, child stunting, wasting, and mortality [28]. Malnutrition in India is contributing to rising cases of nutrition-related diseases, prevalence of anemia among women and children below five years of age (which is currently greater than 50%), child stunting (39%), child underweight (33%), disability, and deaths [29]. Overreliance on cereal-based diets and limited consumption of fruits and vegetables are among the main causes of malnutrition in India [30]. Consumption of fruits and vegetables is 160 and 184g/day in rural and urban India, respectively, which is a far cry from the World Health Organization’s recommendation of a minimum of 400 g/day required for a healthy diet [31].

Women play an important role in agriculture in India. About 55% of economically active women in India are employed in agriculture compared to 40% of men [32]. However, in spite of the significant contribution of women in agriculture, inequalities still exist in terms of their access to assets and productive inputs [33]. Empowerment of women can improve their access to and control over resources in the household and enhance agricultural productivity and nutritional outcomes [10, 14].

Interventions and programs designed to improve women’s empowerment have been rolled out in India. One such program is Mission Shakti (“Shakti” loosely means power in Hindu. Mission Shakti is therefore a mission to empower women), which was launched by the state government of Odisha in 2001 to empower women socially and financially through women’s self-help groups (SHGs) [34]. Another program is the Odisha Particularly Vulnerable Tribal Group Empowerment and Livelihoods Improvement Program (OPELIP). This program has a component that aims to improve women’s empowerment and gender balance in decision-making through the promotion of women SHGs, encouraging the participation of women in local governance, financial literacy, livelihood enhancement, nutrition, basic health care, reproductive health care, women’s rights, entitlements and choices, and rural finance services to enable social and economic development of SHG members [35].

Due to the limited evidence of the effects of women’s empowerment on nutritional outcomes among vulnerable population groups, we conducted our study in the context of OPELIP. OPELIP mainly targeted Scheduled Tribes, which includes Particularly Vulnerable Tribal Groups (PVTGs). PVTGs were of particular interest to the program because they are among the poorest and the most socioeconomically vulnerable and disadvantaged population groups in rural India [35]. India’s tribal population is officially registered according to their distinct cultural and ethnic features called “Scheduled Tribes”. Unlike the Scheduled Castes, which are dispersed throughout the country, Scheduled Tribes are traditionally concentrated in about 15% of the country’s geographical area, and are mainly located in forests, hills, and inaccessible areas [35]. Most of the Scheduled Tribes reside in isolated groups in remote areas with few economic opportunities. Scheduled Tribes are more deprived in food and nutrition security, education, and health compared to any other group in India [30, 35]. For example, Schedule Tribes consume relatively few fruits and vegetables and are thus more vulnerable to malnutrition challenges compared to other population groups in India [30]. Approximately 2.5% of the total Scheduled Tribes’ population belong to the PVTGs [35]. PTVGs is a classification reserved for the most disadvantaged of the Scheduled Tribes’ communities in India. Odisha has the largest number of PVTGs across all Indian states– 13 out of a total of 75 PVTGs distributed across more than 14 states in India are found in Odisha [36, 37]. PVTGs are mostly located in remote areas, are socially marginalized, and are extremely poor and vulnerable to socioeconomic shocks [35].

## Materials and methods

### Study area and sample

This study uses two rounds of panel data collected from a survey of about 1,900 farm households located in 12 districts in the state of Odisha, India. Baseline and mid-term surveys were conducted in June 2017 and November 2021, respectively. Anonymized baseline survey data collected by IFAD was shared with the project team for the research in July 2021. The midline survey was conducted by the research team with funding from IFAD. The farm surveys covered 12 districts of Odisha and 17 micro-project areas (MPAs) where the women’s empowerment program in OPELIP was rolled out. MPAs are government entities/zones (smaller than districts) that were formed in the late 1970s to help with the implementation of special programs targeting PVTGs.

We used a two-stage proportional sampling procedure. In the first stage, proportional stratified sampling was used to determine the number of households to be selected from each of the 17 MPAs in the 12 Districts covered by the program. This was to ensure that there is proportional representation of households from all 17 MPAs and that variation in geographical and agro-climatic conditions in each of the MPAs was captured. In the second stage, the overall target sample of 2,096 households (arrived at following a sample size calculation procedure to achieve 80% statistical power and a 95% confidence level) was divided by 12 (the minimum number of households set to be interviewed in each village). This resulted in the random sampling of 2,096 households from a total 174 villages [38]. There was low random sample attrition of about 8% in the follow-up survey round. S1 Table compares the remaining sample with the attrition sample. Minor differences are observed. However, to further test for possible attrition bias in our analysis, we used inverse probability weighting procedure following Wooldridge [39] and Ogutu *et al*. [40] to estimate the fixed effects regressions described below. In the follow-up survey 1,921 households were surveyed, implying a response rate of 92%. For this analysis, we use balanced panel data of 1,921 households whose data are available in both survey rounds.

### Measurement of dietary diversity and child nutritional outcomes

We want to analyze the effects of women’s empowerment on HDDS, value of home-produced and consumed food per adult equivalent, and child anthropometric measurements. We constructed the HDDS using 7-day food consumption recall data as a simple count of the total food groups consumed by the household in the seven days prior to the survey out of the 12 possible food groups [41]. The HDDS based on 12 food groups is a robust proxy for the access component of food security due to its high positive correlation with calorie intake [42]. We also computed the monthly value of home-produced and consumed food per adult equivalent as an additional proxy for household food availability and consumption using the 7-day food consumption recall data which captured food from own production, purchases, and others (e.g., gifts). The 7-day food consumption figures were multiplied by four to obtain the monthly estimates. Own produced food was valued using prices of food commodities reported at the village level.

To analyze whether women’s empowerment improves child anthropometric measurements, we computed height-for-age z-scores (HAZ), weight-for-age z-scores (WAZ), and weight-for-height z-scores (WHZ) for one child (of age 0–59 months) per household following the World Health Organization’s (2006) growth references. One child was selected per household to allow equal representation of households with children. Limited survey resources also informed the selection of one child. Where more than one child aged 0–59 months resided in the household, the index child was selected at random. HAZ measures chronic undernutrition, while both WAZ and WHZ measure acute or chronic undernutrition among children. From these z-scores, we computed and considered the prevalence of stunting, underweight, or wasting to be present if HAZ, WAZ, and WHZ values were less than two standard deviations from WHO’s growth references [43].

### Measurement of women’s empowerment index

Empowerment is a multidimensional concept that includes three interrelated aspects: (a) resources (access to and future claims to material, human, and social resources); (b) agency (the process of decision-making, negotiation, or bargaining); and (c) achievement (well-being outcomes) [5, 16]. We computed a women’s empowerment index by taking into account these concepts following the Women’s Empowerment in Agriculture Index (WEAI) developed by Alkire *et al*. [44] However, the women’s empowerment index we computed differs from the WEAI as it includes a wider range of 23 decision-making variables rather than the 10 decision variables considered in the WEAI developed by Alkire *et al*. [44].

The 23 intra-household decision-making questions capture the dynamics of decision making in seven decision domains [45]. The decision domains include agricultural input use or production (fertilizer, pesticides, livestock inputs and services, hiring of labor and farm machinery, and asset use or control decisions), crop and livestock sales (crop and livestock purchase and sales decisions), use of cash income or revenue (use of crop, livestock, social transfers, wage, and business incomes), food purchase decisions (small and large food items purchase decisions), non-food purchase decisions (small and large non-food items purchase decisions), child school enrolment, and other decision domain (related to wage employment, credit access and use, and group membership decisions). We also constructed an alternative women’s empowerment index with five decision domains that are more relevant for improved food security and child nutrition. In this restricted index, we excluded questions related to child school enrolment and purchase of non-food items.

Each of the 23 decision variables had three possible responses: the man makes the decision independently; the woman makes the decision independently, or a decision is made together (i.e., a joint decision). However, to focus on women in line with the previous literature which suggest that women are most empowered when they make decisions independently, we reduced the three responses into a binary response with “yes equal to one” if a woman made the decision independently and “no equal to zero” [46, 47]. To construct the women’s empowerment index, we summed the score of all decision variables and divided it by the total number of decisions. This resulted in a women’s empowerment index computed as the share of total decisions made by the woman independently. Just as the WEAI, our index ranges from 0 to 1, with a higher value indicative of a greater degree of women’s empowerment. Using the same approach, we also computed empowerment scores for each empowerment decision domain. As robustness check, we computed women’s empowerment in agriculture index (WEAI1) that considered the woman in a household to be empowered if a woman made decisions alone on selected decision variables following Alkire *et al*. [44]. We relied on the domains, indicators and adequacy cut-offs or weights for WEAI following Alkire et al. [44] as shown in S2 Table. We also computed WEAI2 that considered a household or the woman in a household to be empowered if a woman made decisions alone or jointly.

### Data analysis

Our aim is to evaluate the effects of women’s empowerment on dietary diversity and child nutritional outcomes. Since we use observational data with a higher likelihood of program placement and households self-selecting into the women’s empowerment interventions, identification of the causal effects of the intervention may be challenging because of possible endogeneity problems. However, we have panel data which allows us to observe the sample households across time and reduce potential endogeneity or confounding problems using either random effects (RE) or fixed effects (FE) regression models. RE models assume that unobserved heterogeneity or differences are random and uncorrelated with control variables, but FE models assume that unobserved heterogeneity may be correlated with control variables [39]. Hence, FE models are intuitively more appealing in practice as they help to control for individual or household-level time-invariant heterogeneity. Following Wooldridge [39], we estimate the FE linear regression model as follows:

$$y_{it}=\alpha +\beta {WE}_{it}+{\delta X}_{it}+c_{i}+\upsilon_{it},$$ (1)

where *y*_*it*_ is the HDDS or child anthropometric indicator for household *i* in year *t*, *WE* is the women’s empowerment index computed as the share of total decisions made by the woman independently in household *i* in year *t*, ***X***_*it*_ is a vector of control variables (which includes age of household head, gender of household head, literacy of household head, household size, dependency ratio, farm size, fertilizer use, access to clean water, access to clean fuel/energy, access to clean toilet, and year dummy variables), *c*_*i*_ captures unobserved household-specific effects or differences, and *υ*_*it*_ captures idiosyncratic shocks or (standard) errors clustered at the village level. Consistent with previous studies, we included year dummy variable in the fixed effect regressions to account for temporal variation [48, 49]. The year dummy variable in the fixed effects regression models helps control unobserved factors that could cause differences in the data across the two survey years (i.e., 2017 and 2021), which importantly cover the pre- and earlier post-COVID-19 periods.

We estimated separate models for each of the dietary diversity and child anthropometric indicators discussed above. In all estimated models, the main parameter of interest is *β*, which represents the average effect of women’s empowerment on household dietary diversity and child anthropometry. Apart from the regression models estimated using the FE estimator, we also conducted t-tests to compare: the differences between 2017 and 2021 samples on selected indicators, including dietary and anthropometric indicators; and the differences between households in which women made decisions independently and households in which women did not make decisions independently on selected household characteristics and nutritional outcomes in year 2017 and 2021. All the quantitative analysis were conducted using STATA version 17.

### Ethical considerations

This study was conducted according to the guidelines laid down in the Declaration of Helsinki and all procedures involving research study participants were approved by the Institutional Review Board (IRB) of the Alliance of Bioversity International and CIAT (2021-IRB15). Written or verbal informed consent was obtained from all participants for inclusion in the study.

## Results and discussion

### Descriptive statistics

Table 1 presents the summary statistics of the women’s empowerment indicators. Only 10–12% of the total intra-household decisions were made by women independently in the two survey rounds (about 13% of the decisions were made jointly, while 75% of the decisions were made by men), which suggests a low degree of women’s empowerment. Comparison of the women’s empowerment indicators by year of survey shows significant improvements in 2021 in all the decision domains, except in sales decisions and overall women’s empowerment index. This suggests that existing women empowerment interventions were already increasing women’s empowerment in some decisions by 2021. The contribution of women in each of the decision-making variables also rose in 2021 compared to 2017 (S3 Table). Women’s empowerment was more likely to increase with their age and whether they were the household head but was more likely to decrease with greater household size perhaps due to increased household chores that may reduce their time for participating in women empowerment programs (S4 Table).

**Table 1:** Summary statistics of women’s empowerment indicators.

	(1)	(2)	(3)	(3)-(2)
Variable	Full sample	2017 sample	2021 sample	Mean diff.
	Mean (SD)	Mean (SD)	Mean (SD)	Mean/SD
*Aggregate women’s empowerment indicators*				
Share of women’s solitary decisions (all seven decision domains) ^a^	0.10	0.10	0.11	0.01
	(0.30)	(0.30)	(0.31)	(0.01)
Share of women’s solitary decisions (five nutrition-relevant decision domains) ^b^	0.11	0.10	0.12	0.02**
	(0.30)	(0.29)	(0.31)	(0.01)
*Women’s empowerment decision domains*				
Share of women’s decisions in input use decisions	0.11	0.10	0.11	0.02*
	(0.31)	(0.30)	(0.31)	(0.1)
Share of women’s decisions in sales decisions	0.10	0.10	0.11	0.01
	(0.30)	(0.29)	(0.30)	(0.1)
Share of women’s decisions in income use decisions	0.11	0.09	0.12	0.02**
	(0.30)	(0.29)	(0.31)	(0.1)
Share of women’s decisions in food purchase decisions	0.11	0.10	0.12	0.02**
	(0.31)	(0.30)	(0.32)	(0.1)
Share of women’s decisions in other decisions	0.11	0.10	0.12	0.02**
	(0.30)	(0.29)	(0.31)	(0.1)
Observations	3,842	1,921	1,921	

*Notes*: Mean estimates are shown with standard deviations (SD) in parentheses. ^a^ includes all seven decision domains (input use, sales, income, food purchase, non-food purchase, child schooling, other), ^b^ includes five decision domains relevant for improved nutrition (excludes non-food purchases and child schooling decisions). Mean diff. implies mean difference between 2017 and 2021 sample conducted using t-tests.

* *p* < 0.1

** *p* < 0.05.

Table 2 presents summary statistics of selected socioeconomic variables of the sample households by survey year. Pooled sample results show that on average, household heads were about 47.6 years old, only 12% of the sample households were headed by women. This is consistent with results of Table 1 which show that women made 10⁠–12% of all intra-household decisions independently. At least 80% of household heads were married and more than half were literate (can read and write). On average, households had four adults and a dependency ratio of 0.7. The sample consisted of smallholder farm households with average farm sizes of about 1.8 acres, and around 80% of the farm households used fertilizer. More than three-quarters of the sample had access to safe drinking water and improved energy (e.g., solar, electricity) for home use, but only one-third of households had access to clean toilet facilities, which highlights sanitation problems. Comparison of the selected socioeconomic variables by the decision-maker in the household shows that households with a woman making decisions independently had older household heads, were more likely to be headed by women, were less likely to have a married household head, and had fewer members than households in which decisions were made by men or jointly. Households with a woman making decisions independently were also less likely to use fertilizer or have access to clean toilet facilities as shown by the data in 2017 and 2021. Comparison of the control variables by year of survey (S5 Table) shows a significantly higher value of age of head, increased share of female-headed households, a lower share of married household heads, smaller household sizes, increased land size, a higher share of households using fertilizer, increased access to clean drinking water, better access to sanitary facilities and increased access to improved energy in 2021 compared to 2017. Improvements in clean drinking water, sanitary facilities, and access to energy in 2021 are probably due to the interventions (e.g., OPELIP) rolled out in the study area.

**Table 2:** Summary statistics of selected socioeconomic characteristics.

	(1)	(2)	(3)	(4)	(5)
		2017		2021	
Variable	Pooled sample	Woman does not decide independently	Woman decides independently	Woman does not decide independently	Woman decides independently
	Mean (SD)	Mean (SD)	Mean (SD)	Mean (SD)	Mean (SD)
Age of household head (years)	47.58	46.00	52.03***	48.38	50.21*
	(12.96)	(12.96)	(12.08)	(13.00)	(11.45)
Female head (%)	11.71	0.23	100.00***	3.38	99.51***
	(32.16)	(4.80)	(0.00)	(18.07)	(7.00)
Married head (%)	84.33	94.76	8.11***	91.26	6.37***
	(36.36)	(22.29)	(27.37)	(28.24)	(24.49)
Household head literate (%)	58.30	58.87	55.68	58.65	52.94
	(49.31)	(49.22)	(49.81)	(49.26)	(50.04)
Household size (adult equivalents)	4.04	4.18	3.63***	4.05	3.18***
	(1.52)	(1.51)	(1.71)	(1.48)	(1.54)
Dependency ratio (count)	0.70	0.71	0.68	0.70	0.62
	(0.90)	(0.80)	(1.05)	(0.95)	(1.10)
Land size (acres)	1.79	1.54	1.51	2.12	1.45**
	(3.12)	(2.38)	(2.03)	(3.90)	(1.48)
Household uses fertilizer (%)	82.00	81.61	69.03***	83.64	83.13
	(38.43)	(38.75)	(46.39)	(37.00)	(37.57)
Access to clean drinking water (%)	78.47	75.35	75.14	81.60	81.86
	(41.11)	(43.11)	(43.34)	(38.76)	(38.63)
Access to clean toilet (%)	34.15	29.09	27.03	40.24	32.35**
	(47.43)	(45.43)	(44.53)	(49.05)	(46.90)
Access to improved energy (%)	83.91	75.06	69.73	93.30	93.14
	(36.74)	(43.28)	(46.07)	(25.01)	(25.34)
Observations	3842	1736	185	1717	204

*Notes*: Mean estimates are shown with standard deviations (SD) in parentheses. Mean diff. implies mean difference conducted using t-tests.

* *p* < 0.1

** *p* < 0.05

*** *p* < 0.01.

Table 3 presents summary statistics of HDDS, value of home-produced and consumed foods per adult equivalent, and child anthropometric indicators by decision-maker across the survey years. Pooled sample results show that on average, households consumed about six food groups and home-produced foods worth 137 Indian Rupees per adult male equivalent. Sampled children (0⁠–59 months) had an average HAZ of -1.64 standard deviations, WAZ of -1.63 standard deviations, and WHZ of -0.97 standard deviations. The HAZ, WAZ, and WHZ show that around 45%, 42%, and 20% of the sampled children were stunted, underweight, and wasted, respectively. The higher prevalence rates of child stunting and underweight point at sizeable child nutrition problems among sample households. In both survey rounds, the prevalence of stunting and underweight were higher than those reported by the National Family Health Survey (NFHS) 5, 2019⁠–2021 for the state of Odisha, which shows that between 2019 and 2021, 31% of the children under five years were stunted and 30% were underweight [50]. This is probably because our study focused on PVTGs and scheduled tribes (STs), which are more vulnerable to malnutrition challenges as they tend to consume less nutritious diets. Comparison of the selected socioeconomic variables by the decision-maker in the household shows that in 2021, households with a woman making decisions independently consumed fewer food groups than households in which decisions were made by men or jointly. For all other variables, the differences were not statistically significant.

**Table 3:** Summary statistics of food security and child anthropometric indicators.

	(1)	(2)	(3)	(4)	(5)
		2017		2021	
Variable	Pooled sample	Woman does not decide independently	Woman decides independently	Woman does not decide independently	Woman decides independently
	Mean (SD)	Mean (SD)	Mean (SD)	Mean (SD)	Mean (SD)
*Household food security proxy variables*	5.51	5.83	5.89	5.21	5.05*
Household dietary diversity score (0–12)	(1.47)	(1.60)	(1.66)	(1.24)	(1.23)
Value of home-produced and consumed food per adult equivalent	136.61	120.95	151.11	146.93	169.89
	(235.25)	(254.81)	(305.28)	(189.71)	(314.97)
*Child nutrition variables*					
Height-for-age z-score (HAZ)	-1.64	-1.33	-1.71	-1.84	-1.63
	(1.94)	(2.39)	(1.99)	(1.56)	(1.77)
Prevalence of stunting (%)	45.42	45.20	37.50	45.88	45.16
	(49.82)	(49.86)	(50.00)	(49.89)	(50.59)
Weight-for-age z-score (WAZ)	-1.63	-1.59	-1.37	-1.68	-1.49
	(1.45)	(1.72)	(2.22)	(1.22)	(1.27)
Prevalence of underweight (%)	41.83	43.06	43.75	40.94	41.94
	(49.36)	(49.60)	(51.23)	(49.23)	(50.16)
Weight-for-height z-score (WHZ)	-0.97	-1.14	-0.53	-0.89	-0.69
	(1.44)	(1.71)	(1.91)	(1.22)	(1.24)
Prevalence of wasting (%)	20.85	30.60	25.00	14.35	19.35
	(40.65)	(46.17)	(44.72)	(35.10)	(40.16)
Observations	3842	1736	185	1717	204

*Notes*: Mean estimates are shown with standard deviations (SD) in parentheses. Mean diff. implies mean difference conducted using t-tests.

* *p* < 0.1.

We also compared the summary statistics of HDDS, value of home-produced and consumed foods per adult equivalent, and child anthropometric indicators by survey year to examine possible differences between pre- and early post-COVID-19 period. S6 Table shows mixed results, so it is not possible to clearly attribute the differences to COVID-19. Lower values of HDDS and HAZ were observed in the year 2021 compared to year 2017. However, higher values of home-produced and consumed food per adult equivalent, higher WHZ and reduced prevalence of wasting were observed in in the year 2021 compared to year 2017. A detailed description of all used variables is summarized in S7 Table.

### Regression results

The differences observed in Table 3 cannot be interpreted as causal as they do not control for possible confounding factors. We control for confounding factors (e.g., the differences in various control variables) in the following regression models to net out the effects of women’s empowerment on food consumption, diets, and child anthropometric indicators. The estimation results with inverse probability weighting procedure presented in S8–S11 Tables are similar and consistent with the results without attrition-weighting. This is due to the low random attrition in our sample. Therefore, we will discuss the results without attrition-weighting since attrition did not introduce significant bias in our sample.

Table 4 presents the effects of women’s empowerment on HDDS and value of home-produced and consumed foods per adult equivalent. The fixed effects regression models used fit the data well (*p* < 0.01). Results show that women’s empowerment had a positive and significant effect on HDDS. Women’s empowerment was measured as a continuous variable ranging from zero to one. In this regard, results in Table 4 imply that a 10-percentage point increase in the level of the women’s empowerment index increased the HDDS by 0.04 food groups and the value of home-produced and consumed food per adult equivalent by 37%. Similarly, a 10-percentage point increase in the level of the alternative women’s empowerment index computed with five decisions domains increased the HDDS by 0.07 food groups and value of home-produced and consumed food per adult equivalent by 62%. These are sizeable gains of women’s empowerment on the value of home-produced and consumed food per adult equivalent after controlling for confounding factors. However, the magnitude of the effect of women’s empowerment on the HDDS was relatively small, which suggests that additional interventions may be required to achieve sizeable or higher household dietary diversity. Improved dietary diversity is important as it could contribute to improved child nutrition. Indeed, higher HDDS was associated with lower prevalence of underweight and wasting in children below the age of 5 years as shown in S12 Table.

**Table 4:** Effects of women’s empowerment (share of decisions by women) on HDDS and value of home-produced and consumed foods.

Variable	HDDS	Log of value of home-produced and consumed food	Obs.
Share of decisions by women (0–1) ^a^	0.410*	1.551*	3842
	(0.238)	(0.916)	
Share of decisions by women (0–1) ^b^	0.669**	1.972**	3283
	(0.327)	(0.956)	

*Notes*: HDDS; household dietary diversity score. ^a^ includes all seven decision domains (input use, sales, income, food purchase, non-food purchase, child schooling, other), ^b^ includes five decision domains relevant for improved nutrition (excludes non-food purchase and child schooling decisions). Coefficients are estimated using fixed effects model for panel data and are shown with robust standard errors clustered at the village level in parentheses. Control variables include: age, age of household head, age of head squared, sex of head, marital status of head, literacy of head, household size, dependency ratio, land size, squared land size, fertilizer use, and time.

* *p* < 0.1

** *p* < 0.05.

Table 5 presents the effects of women’s empowerment on child anthropometry. The explanatory variables used in the fixed effects regression models (Tables 5–7) were relevant as was indicated by the F-statistic (which tests if coefficients of explanatory variables are all jointly equal to zero) *p* < 0.01. Results show that a 10-percentage point increase in the level of women’s empowerment significantly increased HAZ, WAZ and WHZ by 0.164, 0.159 and 0.341 standard deviations, respectively. The improvements in z-scores contributed to reduced prevalence of underweight and wasting, but not stunting. For instance, a 10-percentage point increase in the level of women’s empowerment significantly reduced the probability of a child being underweight and wasted by 3.9 and 5.6 percentage points, respectively. These findings imply that a 100% increase in the level of women’s empowerment or complete decision-making power by women in the household with regard to the decisions included in the index would reduce the prevalence of child underweight and wasting by 39 and 56 percentage points, respectively. These are sizeable effects of women’s empowerment on the prevalence of child underweight and wasting. The alternative women’s empowerment index also shows significant increments in WAZ and WHZ, which translate to significant reductions in the prevalence of child underweight and wasting. These findings underline the robustness of this study’s results which suggest that women’s empowerment improves child anthropometry.

**Table 5:** Effects of women’s empowerment (share of decisions by woman alone) on child anthropometry.

Variable	HAZ	Stunting (%)	WAZ	Underweight (%)	WHZ	Wasting (%)	Obs.
Share of decisions by women ^a^	-1.644*	-2.189	1.588***	-38.933**	3.414***	-55.906*	657
	(0.856)	(16.627)	(0.451)	(17.650)	(0.403)	(31.328)	
Share of decisions by women ^b^	-0.852	-16.649	1.899***	-51.476**	3.204***	-60.195**	657
	(0.997)	18.798)	(0.558)	(21.068)	(0.543)	(23.514)	

*Notes*: HAZ; height for age z-score, WAZ; weight for height z-score, WHZ; weight for height z-score. ^a^ includes all seven decision domains (input use, sales, income, food purchase, non-food purchase, child schooling, other), ^b^ includes five decision domains relevant for improved nutrition (excludes non-food purchase and child schooling decisions). Coefficients are estimated using fixed effects model for panel data and are shown with robust standard errors clustered at the village level in parentheses. Control variables include: age, age of household head, age of head squared, sex of head, marital status of head, literacy of head, household size, dependency ratio, land size, squared land size, fertilizer use, time, access to clean water, access to clean fuel/energy, access to clean toilet. We are using panel data with two time periods so the 456 children’s observations should double to 912 if the same children were to be observed in both baseline and follow-up survey rounds. However, some children were not observed in the follow-up survey round (as some had surpassed the age-range of children survey) hence the variation in the children’s observations.

* *p* < 0.1

** *p* < 0.05

*** *p* < 0.01.

**Table 6:** Effect of each decision domain of women’s empowerment (share of decisions by women) on HDDS and value of home-produced and consumed foods.

	(1)	(2)	(3)
Decision domain	HDDS	Log of value of home-produced and consumed food per adult equivalent	Obs.
Agricultural input use	0.409	1.592*	3283
	(0.325)	(0.924)	
Crop and livestock sales	0.695**	1.390	3283
	(0.305)	(0.861)	
Cash income use	0.619*	2.515***	3022
	(0.340)	(0.781)	
Food purchase	0.519*	1.092	3283
	(0.279)	(0.707)	
Other (employment, credit)	0.634**	1.462*	3283
	(0.303)	(0.796)	

*Notes*: HDDS; household dietary diversity score. Coefficients are estimated using fixed effects model for panel data and are shown with robust standard errors clustered at the village level in parentheses. Control variables include: age, age of household head, age of head squared, sex of head, marital status of head, literacy of head, household size, dependency ratio, land size, squared land size, fertilizer use, and time.

* *p* < 0.1

** *p* < 0.05

*** *p* < 0.01.

**Table 7:** Effects of each decision domain of women’s empowerment (share of decisions by women) on child anthropometry.

	(1)	(2)	(3)	(4)	(5)	(6)	(7)
Decision domain	HAZ	Stunting (%)	WAZ	Underweight (%)	WHZ	Wasting (%)	Obs.
Agricultural input use	-1.338	-7.283	1.695***	-43.881**	3.304***	-54.358**	657
	(0.951)	(16.762)	(0.513)	(21.103)	(0.414)	(23.881)	
Crop and livestock sales	-1.319	-10.220	1.608**	-33.839*	3.329***	-41.207*	657
	(1.345)	(28.894)	(0.745)	(18.719)	(0.671)	(24.231)	
Cash income use	-0.977	-24.362	1.948***	-31.212*	3.415***	-33.766*	620
	(1.045)	(22.307)	(0.582)	(18.156)	(0.546)	(20.369)	
Food purchase	-0.305	-24.739	0.924	-14.553	1.454	-25.091	657
	(1.138)	(27.213)	(0.900)	(24.951)	(1.371)	(24.061)	
Other	1.036	-56.765	2.016***	-46.319**	1.912	-32.882	657
	(1.919)	(41.318)	(0.787)	(19.775)	(1.509)	(23.966)	

*Notes*: HAZ; height for age z-score, WAZ; weight for height z-score, WHZ; weight for height z-score. Coefficients are estimated using fixed effects model for panel data and are shown with robust standard errors clustered at the village level in parentheses. Control variables include: age, age of household head, age of head squared, sex of head, marital status of head, literacy of head, household size, dependency ratio, land size, squared land size, fertilizer use, time, access to clean water, access to clean fuel/energy, access to clean toilet.

* *p* < 0.1

** *p* < 0.05

*** *p* < 0.01.

Apart from analyzing the effects of the aggregate women’s empowerment score on HDDS, value of home-produced and consumed foods per adult equivalent, and child anthropometry, we also examined the effects of each of the decision-making domains that make up the aggregate women’s empowerment score. Our aim was to understand which decision-making domains contribute to improved household dietary diversity and child anthropometry. Such an analysis can generate insights for proper targeting of interventions because decision-making domains that matter for improved nutrition may vary depending on the socio-cultural context [24]. In the analysis, we mainly focused on five decision-making domains–agricultural input use or production, sales, income, food purchases, and other (credit, group membership, and employment)–relevant for improved nutrition.

Table 6 presents the effects of the different women’s empowerment decision-making domains on HDDS and value of home-produced and consumed foods per adult equivalent. Column (1) shows that a 10-percentage point increase in the level of women’s empowerment in agricultural sales, income, food purchase, and other (access to credit, group membership and employment) decision-making domains significantly increased HDDS by 0.05–0.07 food groups. These results are plausible since cash income from agricultural sales often plays an important role in improvement of dietary diversity [51, 52]. Column (2) of Table 6 shows that show that a 10-percentage point increase in the level of women’s empowerment in agricultural input use, income, and other (access to credit, group membership and employment) decision-making domains significantly increased the effects on the value of home-produced and consumed foods per adult equivalent by 33%– 113%. Cash income decision-making domain seemed to have greater effects on the value of home-produced and consumed foods per adult equivalent compared to input use and other (employment, group membership and credit access and use) decision-making domains, which underlines the importance of cash income control in household dietary diversity and food availability and consumption.

The results presented in Table 6 are plausible because women’s empowerment in agricultural production and sales decisions is associated with higher levels of technical ability in farming, increased production, and improved household food availability and consumption [16]. Moreover, cash income from agricultural sales tends to contribute to improved household diets by increasing both quantity and diversity of purchased foods more generally, but cash controlled by women is more likely to be spent on food and health care compared to cash controlled by men [53]. Hence, it matters who controls cash income or makes income use decisions within the household. Women farmers often have less access to land, information, capital and credit, and other inputs, which disadvantages them in production compared to male farmers [13]. Therefore, an improvement in women’s decision-making in access to credit and agricultural inputs can enhance their production and improve food security among children in the household.

Table 7 presents the effects of different women’s empowerment decision-making domains on child anthropometry. Consistent with the results of the aggregate women’s empowerment index, the decision-making domains have significant effects on all the anthropometric indicators, except on HAZ and prevalence of stunting. This suggests that in the study context, increasing women’s empowerment in agriculture significantly improves short and medium-term indicators of child nutrition, but it does not have a significant effect on more long-term indicator like stunting, which is a measure of chronic malnutrition. Columns (3) and (4) of Table 7 suggest that a 10-percentage point increase in women’s empowerment in agricultural input use or production, sales, income, food purchase, and other (access to credit, group membership and employment) decision-making domains significantly increased WAZ by 0.169⁠–0.207 standard deviations and reduces the probability of a child being underweight by between 3.1⁠–4.6 percentage points. Women’s empowerment in other (employment, group membership and credit access and use) decision-making domain has a relatively larger effect on WAZ and prevalence of underweight compared to other decision-making domains, which points to the importance of women’s labor contribution, group membership and access to credit in improving child nutrition.

Columns (5) and (6) of Table 7 suggest that a 10-percentage point increase in women’s empowerment in agricultural input use, sales, and income decision-making domain significantly increased WHZ by 0.330–0.342 standard deviations, and reduced the probability of a child being wasted by between 3.4–5.4 percentage points. The results also show that women’s empowerment in income use decisions has a relatively larger effect on WHZ and prevalence of wasting compared to empowerment in other decision-making domains. These results are consistent with results of previous studies which show that greater control of income resources by women can improve child nutritional outcomes [23, 53, 54].

As mentioned, for robustness checks, we computed women’s empowerment index (WEAI1) that considered the women in a household to be empowered if a woman made decisions on selected variables alone. We also computed WEAI2 that considered the women in a household to be empowered if a woman made decisions alone or jointly following Alkire *et al*. [44]. The results of the WEAI1 in S13 Table are consistent with the results of the women’s empowerment index (including 23 decision variables) used in Tables 4 and 5 which show that women’s empowerment improves HDDS, household food availability and consumption (value of home-produced and consumed food), WAZ, and WHZ and reduces the prevalence of underweight and wasting in children below 5 years old. For WEAI2, S14 Table shows that women’s empowerment computed as the share of decisions made by a woman alone or jointly increases HDDS and household food consumption, but it does not influence child anthropometric indicators. These results suggest that women’s empowerment measured by women making decisions alone is better for improvement of child nutrition than women’s empowerment measured by men also contributing to decision-making in our study context. This is consistent with research that showed cash controlled by women is more likely to be spent on food and health care compared to cash controlled by men [53].

## Summary and conclusions

We evaluated the effects of women’s empowerment on household dietary diversity, value of home-produced and consumed foods per adult equivalent (food availability and consumption), and child anthropometry. Results show that women’s empowerment in agriculture is associated with higher household dietary diversity. Sizeable effects of women’s empowerment index on the value of home-produced and consumed foods per adult equivalent were also observed. However, the magnitude of the effect of women’s empowerment on dietary diversity was relatively small, which suggests that complementary interventions may be required to achieve higher household dietary diversity. Our findings are generally consistent with those of previous studies, which showed that women’s empowerment contributes to improved food production and dietary diversity [8, 17, 24].

Women’s empowerment in agricultural sales, income, food purchase, and other (access to credit, group membership and employment) decision-making domains had positive and significant effects on HDDS, while women’s empowerment in agricultural input use, income, and other (access to credit, group membership and employment) decision-making domains had positive and significant effects on the value of home-produced and consumed foods per adult equivalent. These results are plausible because women’s empowerment in agricultural production and sales decisions is associated with higher levels of technical ability in farming, increased production, and improved household diets [16].

Results also showed that women’s empowerment significantly increased WAZ and WHZ but had no effect on HAZ. The improvements in the z-scores contributed to reduced prevalence of underweight and wasting. Analysis of the different women’s empowerment decision-making domains on child anthropometry showed that women’s empowerment in agricultural input use, sales, income, and other (access to credit, group membership and employment) decision-making domains has positive effects on WAZ and reduces the prevalence of underweight. Furthermore, women’s empowerment in agricultural input use, sales, and income decision domain significantly increased WHZ and reduced the probability of a child in the household being wasted. Our findings are consistent with previous studies which showed that greater control of income resources by women can improve child nutritional outcomes [23, 49]. Women are usually the primary caregivers, therefore, their empowerment tends to affect investments in child nutrition, health, and education [12]. Our analysis also showed that not all the decision-making domains had significant effects on all the dietary, food consumption, and child anthropometry indicators examined in this study, which suggests that some decision domains of women’s empowerment may matter more for nutrition than others [18].

Overall, the results showed that women’s empowerment improved household dietary diversity, food consumption, and child anthropometry (reduced prevalence of underweight and wasting in children) among the vulnerable populations in Odisha, India. Hence, empowerment of women may help improve food consumption, household diets, and child nutrition among vulnerable population groups. However, complementary interventions are also needed to address chronic child malnutrition in the study context, since women’s empowerment did not have an effect on the prevalence of child stunting. Empowerment of women in agricultural input use or production, sales, income, food purchase, and other (employment, group membership and credit access) decisions were shown to be important for food consumption, dietary diversity and child nutrition. Hence, strengthening women’s empowerment in these decision domains through promotion of women’s access to land, information, capital and credit, and other inputs, which often disadvantage them in agriculture compared to men, can contribute to improved diets and child nutrition in smallholder farm households.

Most of the PVTGs and other vulnerable population groups often reside in remote areas with poor infrastructure and limited economic opportunities as was evident in our study context [35]. Therefore, to achieve greater impact, programs that aim to improve women’s empowerment may need to be accompanied with improvements in infrastructure to facilitate access to input and output markets, credit facilities, agricultural information (e.g., extension services), land tenure security and off-farm employment opportunities. Our study provides evidence that improvements in women’s social status through decision-making that gives them access to and control over resources can improve nutrition. It adds to the limited evidence supporting the strength of such mechanisms, especially in agriculture and nutrition-related programs [6, 15, 16]. The evidence of the effects of women’s empowerment on dietary diversity and child anthropometry among the vulnerable populations groups in India is crucial. However, the number of respondents for our data on child anthropometry was relatively small. Future studies could provide additional insights by examining the effects of women’s empowerment on child anthropometry using larger panel datasets with vulnerable population groups in other geographical contexts.

## Supporting information

S1 Table Attrition balance table. Summary statistics selected socioeconomic characteristics at baseline for non-attrition and attrition samples.
(DOCX)

S2 Table The domains, indicators and adequacy cut-offs or weights for women’s empowerment in agriculture index. (DOCX)

S3 Table Percent (%) of women’s contribution in each decision variable. (DOCX)

S4 Table Correlation coefficient of between women’s empowerment and selected socioeconomic variables. (DOCX)

S5 Table Comparison of selected socioeconomic characteristics by year of survey. (DOCX)

S6 Table Comparison of food security and child anthropometric indicators by year of survey. (DOCX)

S7 Table Summary and description of variables used in the analysis. (DOCX)

S8 Table Effects of women’s empowerment (share of decisions by women) on HDDS and value of home-produced and consumed foods–attrition-weighted results. (DOCX)

S9 Table Effects of women’s empowerment (share of decisions by women) on child anthropometry–attrition-weighted results. (DOCX)

S10 Table Effect of each decision domain of women’s empowerment (share of decisions by women) on HDDS and value of home-produced and consumed foods–attrition-weighted results. (DOCX)

S11 Table Effects of each decision domain of women’s empowerment (share of decisions by women) on child anthropometry–attrition-weighted results. (DOCX)

S12 Table Association between dietary diversity and child anthropometry. (DOCX)

S13 Table Effects of women’s empowerment (share of decisions made by women alone) on dietary diversity, value food produced and consumed and child anthropometry. (DOCX)

S14 Table Effects of women’s empowerment (share of decisions made by women or jointly) on dietary diversity, value food produced and consumed and child anthropometry. (DOCX)
