# Supplementary material for: Women’s empowerment, household dietary diversity, and child anthropometry among vulnerable populations in Odisha, India
Source: PLoS One. 2024 Aug 6;19(8):e0305204. doi: 10.1371/journal.pone.0305204 (PMC11302906; doi:10.1371/journal.pone.0305204)
Supplement: S1 Table — Summary statistics selected socioeconomic characteristics at baseline for non-attrition and attrition samples. (DOCX) [file pone.0305204.s001.docx]

**S1 Table**. Attrition balance table. Summary statistics selected socioeconomic characteristics at baseline for non-attrition and attrition samples.

|  |  | (1) | (2) |
| --- | --- | --- | --- |
| Variable |  | Retained sample | Attrition sample |
|  |  | Mean (SD) | Mean (SD) |
| Age of household head (years) |  | 46.58 | 43.58 |
|  |  | (12.99) | (13.23) |
| Female head (%) |  | 9.83 | 10.67 |
|  |  | (29.79) | (30.97) |
| Married head (%) |  | 86.41 | 84.27 |
|  |  | (34.27) | (36.51) |
| Household head literate (%) |  | 60.28 | 55.06 |
|  |  | (48.94) | (49.88) |
| Household size (adult equivalents) |  | 4.12 | 3.69 |
|  |  | (1.53) | (1.41) |
| Dependency ratio (count) |  | 0.66 | 0.66 |
|  |  | (0.64) | (0.64) |
| Land size (acres) |  | 1.54 | 1.02 |
|  |  | (2.35) | (1.03) |
| Household uses fertilizer (%) |  | 80.43 | 80.42 |
|  |  | (39.68) | (39.82) |
| Access to clean drinking water (%) |  | 75.32 | 71.91 |
|  |  | (43.12) | (45.08) |
| Access to clean toilet (%) |  | 28.89 | 16.85 |
|  |  | (45.33) | (37.54) |
| Access to improved energy (%) |  | 74.54 | 66.29 |
|  |  | (43.57) | (47.40) |
| Observations |  | 1921 | 178 |

*Notes*: Mean estimates are shown with standard deviations (SD) in parentheses.
