# Supplementary material for: Women’s empowerment, household dietary diversity, and child anthropometry among vulnerable populations in Odisha, India
Source: PLoS One. 2024 Aug 6;19(8):e0305204. doi: 10.1371/journal.pone.0305204 (PMC11302906; doi:10.1371/journal.pone.0305204)
Supplement: S2 Table — (DOCX) [file pone.0305204.s002.docx]

**S2 Table.** The domains, indicators and adequacy cut-offs or weights for Women’s Empowerment in Agriculture Index

| **Domain** | **Indicators** | **Adequacy threshold or cut-off** | **Weight** |
| --- | --- | --- | --- |
| Production | Input in productive decisions | Woman decides the use of fertilizer, either solely or jointly with spouse/man | 1/10 |
|  | Autonomy in production | Woman decides crop/livestock production, either solely or jointly | 1/10 |
| Resources | Ownership of assets | Woman decides the purchase of farm assets, either solely or jointly | 1/15 |
|  | Purchase of assets | Woman decides the purchase of non-farm assets, either solely or jointly | 1/15 |
|  | Access to credit | Woman decides access to credit, either solely or jointly | 1/15 |
| Income | Control over use of income | Woman decides or controlled use of income, either solely or jointly | 1/5 |
| leadership | Group membership | Woman was a member of a group, either solely or jointly | 1/5 |
| Time | Worked load | Woman decided to reduce workload by hiring labor, either solely or jointly | 1/5 |
