# Supplementary material for: Women’s empowerment, household dietary diversity, and child anthropometry among vulnerable populations in Odisha, India
Source: PLoS One. 2024 Aug 6;19(8):e0305204. doi: 10.1371/journal.pone.0305204 (PMC11302906; doi:10.1371/journal.pone.0305204)
Supplement: S3 Table — (DOCX) [file pone.0305204.s003.docx]

**S3 Table.** Percent (%) of women’s contribution in each decision variable.

|  | Decision maker | Child schooling | Fertilizer use | Pesticide use | Hiring labor | Machinery use | Livestock production | Purchase of small food items | Purchase of large food items | Purchase of small non-food items | Purchase of large non-food items |
| --- | --- | --- | --- | --- | --- | --- | --- | --- | --- | --- | --- |
| Year 2017 | Female only | 9.68 | 9.74 | 9.69 | 9.74 | 9.74 | 9.38 | 9.79 | 9.79 | 9.58 | 9.68 |
|  | Male only | 87.56 | 88.33 | 88.22 | 88.33 | 88.33 | 88.18 | 87.35 | 87.30 | 85.83 | 86.93 |
|  | Joint | 2.76 | 1.93 | 2.09 | 1.93 | 1.93 | 2.44 | 2.86 | 2.92 | 4.58 | 3.38 |
|  | % of women making decisions in variable | 12.44 | 11.67 | 11.78 | 11.67 | 11.67 | 11.82 | 12.65 | 12.71 | 14.16 | 13.06 |
|  | Observations | 1921 | 1919 | 1919 | 1919 | 1919 | 1760 | 1921 | 1921 | 1921 | 1921 |
|  |  |  |  |  |  |  |  |  |  |  |  |
| Year 2021 | Female only | 11.19 | 11.04 | 10.88 | 10.98 | 11.00 | 11.40 | 12.08 | 11.82 | 11.04 | 11.04 |
|  | Male only | 52.21 | 66.70 | 66.27 | 66.63 | 66.74 | 55.61 | 54.66 | 52.84 | 54.55 | 53.20 |
|  | Joint | 36.60 | 22.25 | 22.85 | 22.39 | 22.26 | 32.99 | 33.26 | 35.35 | 34.41 | 35.76 |
|  | % of women in decision variable | 47.79 | 33.29 | 33.73 | 33.37 | 33.26 | 44.39 | 45.34 | 47.17 | 45.45 | 46.80 |
|  | Observations | 1921 | 1829 | 1829 | 1831 | 1828 | 1649 | 1921 | 1921 | 1921 | 1921 |

|  | Decision maker | Credit application | Use of business income | Employment decision | Use of wage income | Use of income transfers | Sale or purchase of livestock | Use of livestock income | Purchase of farm assets | Sale of non-farm assets | Use of loan |
| --- | --- | --- | --- | --- | --- | --- | --- | --- | --- | --- | --- |
| Year 2017 | Female only | 9.69 | 10.92 | 9.75 | 9.75 | 15.60 | 8.75 | 5.62 | 9.36 | 9.05 | 8.13 |
|  | Male only | 86.93 | 86.83 | 86.62 | 84.13 | 78.72 | 88.70 | 93.82 | 88.20 | 88.12 | 87.95 |
|  | Joint | 3.39 | 2.24 | 3.63 | 6.12 | 5.67 | 2.55 | 0.56 | 2.45 | 2.83 | 3.92 |
|  | % of women in decision variable | 13.08 | 13.16 | 13.38 | 15.87 | 21.27 | 11.30 | 6.18 | 11.81 | 11.88 | 12.5 |
|  | Observations | 1920 | 357 | 882 | 882 | 141 | 1726 | 178 | 1881 | 1734 | 332 |
|  |  |  |  |  |  |  |  |  |  |  |  |
| Year 2021 | Female only | 11.56 | 8.31 | 10.81 | 10.18 | 13.10 | 11.02 | 10.98 | 11.24 | 8.79 | 9.98 |
|  | Male only | 56.43 | 51.44 | 41.53 | 40.63 | 52.57 | 49.73 | 52.55 | 58.64 | 48.79 | 54.50 |
|  | Joint | 32.01 | 40.26 | 48.29 | 49.19 | 34.33 | 39.25 | 36.47 | 30.12 | 42.41 | 35.52 |
|  | % of women in decision variable | 43.57 | 48.57 | 52.34 | 59.37 | 47.43 | 50.27 | 47.45 | 41.36 | 51.2 | 45.5 |
|  | Observations | 1921 | 313 | 1109 | 1109 | 1304 | 1650 | 1647 | 1868 | 1864 | 441 |

|  | Decision maker | Membership farmer groups | Use of crop income | Crop sale decision |
| --- | --- | --- | --- | --- |
| Year 2017 | Female only | 9.58 | 9.81 | 9.80 |
|  | Male only | 85.32 | 86.85 | 86.76 |
|  | Joint | 5.10 | 3.34 | 3.43 |
|  | % of women in decision variable | 14.68 | 13.15 | 13.23 |
|  | Observations | 1921 | 1020 | 1020 |
|  |  |  |  |  |
| Year 2021 | Female only | 11.43 | 10.52 | 10.33 |
|  | Male only | 48.08 | 67.90 | 67.85 |
|  | Joint | 40.49 | 21.59 | 21.82 |
|  | % of women in decision variable | 51.92 | 32.11 | 32.15 |
|  | Observations | 1921 | 1625 | 1626 |
