# Supplementary material for: Women’s empowerment, household dietary diversity, and child anthropometry among vulnerable populations in Odisha, India
Source: PLoS One. 2024 Aug 6;19(8):e0305204. doi: 10.1371/journal.pone.0305204 (PMC11302906; doi:10.1371/journal.pone.0305204)
Supplement: S4 Table — (DOCX) [file pone.0305204.s004.docx]

**S4 Table.** Correlation coefficient of between women’s empowerment and selected socioeconomic variables.

|  | Age of woman | Household size | Household head vs Other | Spouse vs Other |
| --- | --- | --- | --- | --- |
| Share of women's solitary decisions (all seven decision domains) ^a^ | 0.002^***^ | -0.026^***^ | 0.721^***^ | -0.129^***^ |
|  | (0.000) | (0.003) | (0.012) | (0.006) |
| Obs. | 5827 | 5827 | 5827 | 5827 |

Coefficients are estimated using ordinary least square regression models and are shown with standard errors in parentheses. ^***^ *p* < 0.01.
