# Supplementary material for: Women’s empowerment, household dietary diversity, and child anthropometry among vulnerable populations in Odisha, India
Source: PLoS One. 2024 Aug 6;19(8):e0305204. doi: 10.1371/journal.pone.0305204 (PMC11302906; doi:10.1371/journal.pone.0305204)
Supplement: S5 Table — (DOCX) [file pone.0305204.s005.docx]

**S5 Table**. Comparison of selected socioeconomic characteristics by year of survey

|  | (1) | (2) | (3) |
| --- | --- | --- | --- |
| Variable | Pooled sample | 2017 | 2021 |
|  | Mean (SD) | Mean (SD) | Mean (SD) |
| Age of head (years) | 47.58 | 46.59 | 48.57^***^ |
|  | (12.96) | (13.00) | (12.85) |
| Female head (%) | 11.71 | 9.84 | 13.59^***^ |
|  | (32.16) | (29.79) | (34.27) |
| Married head (%) | 84.33 | 86.41 | 82.25^***^ |
|  | (36.36) | (34.27) | (38.22) |
| Literate (%) | 58.30 | 58.56 | 58.04 |
|  | (49.31) | (49.27) | (49.36) |
| Adult equivalent size (adult equivalents) | 4.04 | 4.12 | 3.96^***^ |
|  | (1.52) | (1.54) | (1.51) |
| Dependency ratio (count) | 0.70 | 0.71 | 0.70 |
|  | (0.90) | (0.83) | (0.97) |
| Land size (acres) | 1.79 | 1.54 | 2.05^***^ |
|  | (3.12) | (2.35) | (3.72) |
| Household uses fertilizer (%) | 82.00 | 80.43 | 83.59^**^ |
|  | (38.43) | (39.68) | (37.05) |
| Access to clean drinking water (%) | 78.47 | 75.33 | 81.62^***^ |
|  | (41.11) | (43.12) | (38.74) |
| Access to clean toilet (%) | 34.15 | 28.89 | 39.41^***^ |
|  | (47.43) | (45.34) | (48.88) |
| Access to improved energy (%) | 83.91 | 74.54 | 93.28^***^ |
|  | (36.74) | (43.57) | (25.04) |
| Observations | 3842 | 1921 | 1921 |

*Notes*: Mean estimates are shown with standard deviations (SD) in parentheses. Mean diff. implies mean difference conducted using t-tests. ^**^ *p* < 0.05, ^***^ *p* < 0.01.
