# Supplementary material for: Women’s empowerment, household dietary diversity, and child anthropometry among vulnerable populations in Odisha, India
Source: PLoS One. 2024 Aug 6;19(8):e0305204. doi: 10.1371/journal.pone.0305204 (PMC11302906; doi:10.1371/journal.pone.0305204)
Supplement: S6 Table — (DOCX) [file pone.0305204.s006.docx]

**S6 Table**. Comparison of food security and child anthropometric indicators by year of survey

|  | (1) | (2) | (3) |
| --- | --- | --- | --- |
| Variables | Pooled sample | 2017 | 2021 |
|  | Mean (SD) | Mean (SD) | Mean (SD) |
| Household dietary diversity score (0–12) | 5.51 | 5.84 | 5.19^***^ |
|  | (1.47) | (1.61) | (1.24) |
| Value of home-produced and consumed food per adult equivalent | 136.61 | 123.85 | 149.37^***^ |
|  | (235.25) | (260.16) | (206.65) |
| *Child nutrition variables* |  |  |  |
| Height-for-age z-score (HAZ) | -1.64 | -1.35 | -1.82^***^ |
|  | (1.94) | (2.37) | (1.57) |
| Prevalence of stunting (%) | 45.42 | 44.78 | 45.83 |
|  | (49.82) | (49.81) | (49.88) |
| Weight-for-age z-score (WAZ) | -1.63 | -1.58 | -1.66 |
|  | (1.45) | (1.74) | (1.22) |
| Prevalence of underweight (%) | 41.83 | 43.10 | 41.01 |
|  | (49.36) | (49.60) | (49.24) |
| Weight-for-height z-score (WHZ) | -0.97 | -1.11 | -0.87^**^ |
|  | (1.44) | (1.72) | (1.22) |
| Prevalence of wasting (%) | 20.85 | 30.30 | 14.69^***^ |
|  | (40.65) | (46.03) | (35.44) |
| Observations | 3842 | 1921 | 1921 |

*Notes*: Mean estimates are shown with standard deviations (SD) in parentheses. Mean diff. implies mean difference conducted using t-tests.  ^**^ *p* < 0.05, ^***^ *p* < 0.01.
