# Supplementary material for: Women’s empowerment, household dietary diversity, and child anthropometry among vulnerable populations in Odisha, India
Source: PLoS One. 2024 Aug 6;19(8):e0305204. doi: 10.1371/journal.pone.0305204 (PMC11302906; doi:10.1371/journal.pone.0305204)
Supplement: S7 Table — (DOCX) [file pone.0305204.s007.docx]

**S7 Table**. Summary and description of variables used in the analysis.

| Variable | Description |
| --- | --- |
| **Panel 1: women’s empowerment indicators** |  |
| *Aggregate women’s empowerment indicators* |  |
| Share (proportion) of women's solitary decisions (all seven decision domains) ^a^ | Total share of women's solitary decisions (in all seven decision domains). Fraction ranging from 0-1. |
| Share of women's solitary decisions (five nutrition-relevant decision domains) ^b^ | Total share of women's solitary decisions (in five nutrition-relevant decision domains). Fraction ranging from 0-1. |
| WEAI1 | Total share of decisions a woman makes alone on a selected number of decision variables shown in S2 Table. Cutt-off weights are used as shown S2 Table. |
| WEAI2 | Total share of decisions a woman makes alone or jointly on a selected number of decision variables shown in S2 Table. Cutt-off weights are used as shown S2 Table. |
| *Women’s empowerment decision domain indicators* |  |
| Share of women’s decisions in input use decisions | Total share of women’s solitary decisions in input use decisions. Fraction ranging from 0-1. |
| Share of women’s decisions in sales decisions | Total share of women’s solitary decisions in sales decisions. Fraction ranging from 0-1. |
| Share of women’s decisions in income use decisions | Total share of women’s solitary decisions in income use decisions. Fraction ranging from 0-1. |
| Share of women’s decisions in food purchase decisions | Total share of women’s solitary decisions in food purchase decisions. Fraction ranging from 0-1. |
| Share of women’s decisions in other decisions | Total share of women’s solitary decisions in other decisions. Fraction ranging from 0-1. |
| **Panel 2: Selected control variables** |  |
| Age of head (years) | Age of household head in years |
| Female head (%) | Household is headed by woman (yes=100, No =0) |
| Married head (%) | Household head is married (yes=100, No =0) |
| Literate (%) | Household head can read and write (yes=100, No =0) |
| Adult equivalent size (adult equivalents) | Household size based on adult equivalents. Household size is computed considering differences in age and gender and energy requirements. |
| Dependency ratio (count) | Dependency ratio is computed as the number of dependents aged zero to 14 and over the age of 65, compared with the total population aged 15 to 64 |
| Land size (acres) | Total cultivated land size in acres |
| Household uses fertilizer (%) | Household uses fertilizer (yes=100, No =0) |
| Access to clean drinking water (%) | Access to clean drinking water (yes=100, No =0) |
| Access to clean toilet (%) | Access to clean toilet (yes=100, No =0) |
| Access to improved energy (%) | Access to improved energy (yes=100, No =0) |
|  |  |
| **Panel 3: Selected dietary diversity, food consumption and anthropometric indicators** |  |
| Household dietary diversity score (0–12) | Simple count of food groups consumed by the household in the last 7 days, ranges from 0-12 food groups. |
| Value of home-produced and consumed food per adult equivalent | Value of home-produced and consumed food per adult equivalent in Indian rupees per month. The 7-day food consumption figures were multiplied by four to obtain the monthly estimates. |
|  |  |
|  |  |
| *Child nutrition variables* |  |
| Height-for-age z-score (HAZ) | Height-for-age z-score |
| Prevalence of stunting (%) | Child has height-for-age z-score of less than -2 standard deviations from WHO growth references (yes=100, No =0) |
| Weight-for-age z-score (WAZ) | Weight-for-age z-score |
| Prevalence of underweight (%) | Child has weight-for-age z-score of less than -2 standard deviations from WHO growth references (yes=100, No =0) |
| Weight-for-height z-score (WHZ) | Weight-for-height z-score (WHZ) |
| Prevalence of wasting (%) | Child has weight-for-height z-score of less than -2 standard deviations from WHO growth references (yes=100, No =0) |

*Notes*: ^a^ includes all seven decision domains (input use, sales, income, food purchase, non-food purchase, child schooling, other), ^b^ includes five decision domains relevant for improved nutrition (excludes non-food purchases and child schooling decisions).
