# Supplementary material for: Women’s empowerment, household dietary diversity, and child anthropometry among vulnerable populations in Odisha, India
Source: PLoS One. 2024 Aug 6;19(8):e0305204. doi: 10.1371/journal.pone.0305204 (PMC11302906; doi:10.1371/journal.pone.0305204)
Supplement: S8 Table — (DOCX) [file pone.0305204.s008.docx]

**S8 Table**. Effects of women’s empowerment (share of decisions by women) on HDDS and value of home-produced and consumed foods – attrition-weighted results.

| Variable | HDDS | Log of value of home-produced and consumed food | Obs. |
| --- | --- | --- | --- |
| Share of decisions by women (0-1) ^a^ | 0.951^***^ | 1.761^*^ | 3842 |
|  | (0.288) | (0.997) |  |
| Share of decisions by women (0-1) ^b^ | 0.639^*^ | 2.270^**^ | 3283 |
|  | (0.361) | (1.062) |  |

*Notes*: HDDS; household dietary diversity score. ^a^ includes all seven decision domains (input use, sales, income, food purchase, non-food purchase, child schooling, other), ^b^ includes five decision domains relevant for improved nutrition (excludes non-food purchase and child schooling decisions). Coefficients are estimated using fixed effects model for panel data and are shown with robust standard errors clustered at the village level in parentheses. Control variables include age, age of household head, age of head squared, sex of head, marital status of head, literacy of head, household size, dependency ratio, land size, squared land size, fertilizer use, and time. ^*^ *p* < 0.1, ^**^ *p* < 0.05, ^***^ *p* < 0.01.
