# Supplementary material for: Women’s empowerment, household dietary diversity, and child anthropometry among vulnerable populations in Odisha, India
Source: PLoS One. 2024 Aug 6;19(8):e0305204. doi: 10.1371/journal.pone.0305204 (PMC11302906; doi:10.1371/journal.pone.0305204)
Supplement: S9 Table — (DOCX) [file pone.0305204.s009.docx]

**S9 Table**. Effects of women’s empowerment (share of decisions by women) on child anthropometry – attrition-weighted results.

| Variable | HAZ | Stunting (%) | WAZ | Underweight (%) | WHZ | Wasting (%) | Obs. |
| --- | --- | --- | --- | --- | --- | --- | --- |
| Share of decisions by women ^a^ | -1.838^*^ | 6.462 | 1.580^***^ | -50.522^***^ | 3.393^***^ | -69.445^**^ | 657 |
|  | (0.997) | (18.931) | (0.48) | (18.165) | (0.502) | (33.414) |  |
| Share of decisions by women ^b^ | -0.918 | -4.246 | 1.838^***^ | -58.584^***^ | 3.150^***^ | -65.291^**^ | 657 |
|  | (1.120) | (20.132) | (0.634) | (21.188) | (0.482) | (27.348) |  |

*Notes*: HAZ; height for age z-score, WAZ; weight for height z-score, WHZ; weight for height z-score. ^a^ includes all seven decision domains (input use, sales, income, food purchase, non-food purchase, child schooling, other), ^b^ includes five decision domains relevant for improved nutrition (excludes non-food purchase and child schooling decisions). Coefficients are estimated using fixed effects model for panel data and are shown with robust standard errors clustered at the village level in parentheses. Control variables include age, age of household head, age of head squared, sex of head, marital status of head, literacy of head, household size, dependency ratio, land size, squared land size, fertilizer use, time, access to clean water, access to clean fuel/energy, access to clean toilet. We are using panel data with two time periods so the 456 children’s observations should double to 912 if the same children were to be observed in both baseline and follow-up survey rounds. However, some children were not observed in the follow-up survey round (as some had surpassed the age-range of children survey) hence the variation in the children’s observations. ^*^ *p* < 0.1, ^**^ *p* < 0.05, ^***^ *p* < 0.01.
