# Supplementary material for: Women’s empowerment, household dietary diversity, and child anthropometry among vulnerable populations in Odisha, India
Source: PLoS One. 2024 Aug 6;19(8):e0305204. doi: 10.1371/journal.pone.0305204 (PMC11302906; doi:10.1371/journal.pone.0305204)
Supplement: S10 Table — (DOCX) [file pone.0305204.s010.docx]

**S10 Table**. Effect of each decision domain of women’s empowerment (share of decisions by women) on HDDS and value of home-produced and consumed foods – attrition-weighted results.

|  | (1) | (2) | (3) |
| --- | --- | --- | --- |
| Decision domain | HDDS | Log of value of home-produced and consumed food per adult equivalent | Obs. |
| Agricultural input use | 0.460 | 1.842^*^ | 3283 |
|  | (0.345) | (1.006) |  |
| Crop and livestock sales | 0.646^*^ | 1.887^**^ | 3283 |
|  | (0.354) | (0.956) |  |
| Cash income use | 0.648 | 2.891^***^ | 3022 |
|  | (0.418) | (0.833) |  |
| Food purchase | 0.562^**^ | 1.396^*^ | 3283 |
|  | (0.255) | (0.755) |  |
| Other (employment, credit) | 0.696^**^ | 1.726^**^ | 3283 |
|  | (0.321) | (0.874) |  |

*Notes*: HDDS; household dietary diversity score. Coefficients are estimated using fixed effects model for panel data and are shown with robust standard errors clustered at the village level in parentheses. Control variables include age, age of household head, age of head squared, sex of head, marital status of head, literacy of head, household size, dependency ratio, land size, squared land size, fertilizer use, and time. ^*^ *p* < 0.1, ^**^ *p* < 0.05, ^***^ *p* < 0.01.

.
