# Supplementary material for: Women’s empowerment, household dietary diversity, and child anthropometry among vulnerable populations in Odisha, India
Source: PLoS One. 2024 Aug 6;19(8):e0305204. doi: 10.1371/journal.pone.0305204 (PMC11302906; doi:10.1371/journal.pone.0305204)
Supplement: S11 Table — (DOCX) [file pone.0305204.s011.docx]

**S11 Table**. Effects of each decision domain of women’s empowerment (share of decisions by women) on child anthropometry – attrition-weighted results.

|  | (1) | (2) | (3) | (4) | (5) | (6) | (7) |
| --- | --- | --- | --- | --- | --- | --- | --- |
| Decision domain | HAZ | Stunting (%) | WAZ | Underweight (%) | WHZ | Wasting (%) | Obs. |
| Agricultural input use | -1.182 | 0.472 | 1.737^***^ | -54.724^***^ | 3.220^***^ | -62.748^**^ | 657 |
|  | (1.095) | (19.552) | (0.608) | (20.573) | (0.450) | (23.881) |  |
| Crop and livestock sales | -1.423 | -1.716 | 1.478^*^ | -40.375^*^ | 3.130^***^ | -39.392 | 657 |
|  | (1.579) | (29.676) | (0.789) | (22.078) | (0.639) | (29.912) |  |
| Cash income use | -1.004 | -16.529 | 1.753^***^ | -39.143^*^ | 3.218^***^ | -39.709^*^ | 620 |
|  | (1.279) | (23.923) | (0.657) | (21.451) | (0.573) | (23.680) |  |
| Food purchase | -0.961 | -6.791 | 1.241 | -33.557 | 2.379^***^ | -33.291 | 657 |
|  | (1.455) | (28.278) | (0.787) | (21.558) | (0.842) | (28.012) |  |
| Other | 0.096 | -28.447 | 1.706^**^ | -48.250^**^ | 2.192^**^ | -34.259 | 657 |
|  | (1.674) | (32.861) | (0.794) | (22.106) | (1.086) | (28.671) |  |

*Notes*: HAZ; height for age z-score, WAZ; weight for height z-score, WHZ; weight for height z-score. Coefficients are estimated using fixed effects model for panel data and are shown with robust standard errors clustered at the village level in parentheses. Control variables include age, age of household head, age of head squared, sex of head, marital status of head, literacy of head, household size, dependency ratio, land size, squared land size, fertilizer use, time, access to clean water, access to clean fuel/energy, access to clean toilet. ^*^ *p* < 0.1, ^**^ *p* < 0.05, ^***^ *p* < 0.01.
