# Supplementary material for: Women’s empowerment, household dietary diversity, and child anthropometry among vulnerable populations in Odisha, India
Source: PLoS One. 2024 Aug 6;19(8):e0305204. doi: 10.1371/journal.pone.0305204 (PMC11302906; doi:10.1371/journal.pone.0305204)
Supplement: S12 Table — (DOCX) [file pone.0305204.s012.docx]

**S12 Table**. Association between dietary diversity and child anthropometry.

| Variable | HAZ | Stunting (%) | WAZ | Underweight (%) | WHZ | Wasting (%) |
| --- | --- | --- | --- | --- | --- | --- |
| HDDS | 0.003 | -1.838 | 0.075^**^ | -2.098^**^ | 0.101^***^ | -3.039^***^ |
|  | (0.049) | (1.276) | (0.037) | (1.231) | (0.039) | (1.057) |
| Obs. | 657 | 657 | 657 | 657 | 657 | 657 |

*Notes*: HDDS; household dietary diversity score, HAZ; height for age z-score, WAZ; weight for height z-score, WHZ; weight for height z-score. Coefficients are estimated using panel data regressions are shown with robust standard errors in parentheses. Control variables include: age, age of household head, marital status of head, literacy of head, household size, dependency ratio, land size, time, access to clean water, access to clean toilet. ^*^ *p* < 0.1, ^**^ *p* < 0.05, ^***^ *p* < 0.01.
