# Supplementary material for: Women’s empowerment, household dietary diversity, and child anthropometry among vulnerable populations in Odisha, India
Source: PLoS One. 2024 Aug 6;19(8):e0305204. doi: 10.1371/journal.pone.0305204 (PMC11302906; doi:10.1371/journal.pone.0305204)
Supplement: S14 Table — (DOCX) [file pone.0305204.s014.docx]

**S14 Table**. Effects of women’s empowerment (share of decisions made by women or jointly) on dietary diversity, value food produced and consumed and child anthropometry.

| Variable | HDDS | Value of home-produced and consumed food | HAZ | Stunting (%) | WAZ | Underweight (%) | WHZ | Wasting (%) |
| --- | --- | --- | --- | --- | --- | --- | --- | --- |
| WEAI2 (0-1) ^a^ | 0.731^***^ | 54.935^*^ | -1.455 | 9.027 | -0.451 | 5.353 | 0.701 | 1.269 |
|  | (0.141) | (32.435) | (1.077) | (32.215) | (0.671) | (19.999) | (0.588) | (18.034) |
| *Attrition-weighted results* |  |  |  |  |  |  |  |  |
| WEAI2 (0-1) ^a^ | 1.790^***^ | 100.320^***^ | -1.592 | 15.927 | -0.444 | 9.605 | 0.808 | -5.338 |
|  | (0.195) | (31.819) | (1.378) | (37.496) | (0.754) | (26.380) | (0.623) | (17.457) |
| Obs. | 3284 | 3284 | 657 | 657 | 657 | 657 | 657 | 657 |

*Notes*: **^a^** Total share of decisions a woman makes alone or jointly on selected decision variables. HDDS; household dietary diversity score. HAZ; height for age z-score, WAZ; weight for height z-score, WHZ; weight for height z-score. Coefficients are estimated using fixed effects model for panel data and are shown with robust standard errors clustered at the village level in parentheses. Control variables include age, age of household head, age of head squared, sex of head, marital status of head, literacy of head, household size, dependency ratio, land size, squared land size, fertilizer use, time, access to clean water, access to clean fuel/energy, access to clean toilet. * p < 0.1, ** p < 0.05, *** p < 0.01.
